# Pd/ZnO nanorods based sensor for highly selective detection of extremely low concentration hydrogen

**DOI:** 10.1038/s41598-017-00362-x

**Published:** 2017-03-22

**Authors:** Mohit Kumar, Vijendra Singh Bhati, Sapana Ranwa, Jitendra Singh, Mahesh kumar

**Affiliations:** 1 Department of Electrical Engineering, Indian Institute of Technology Jodhpur, Jodhpur, 342011 India; 2 Department of Physics, Indian Institute of Technology Jodhpur, Jodhpur, 342011 India; 3 Sensors & Nanotechnology Group, CSIR-Central Electronics Engineering Research Institute, Pilani, Rajasthan 333031 India

## Abstract

We report highly hydrogen selective Pd contacted ZnO nanorods based sensor detecting low concentration even at low operating temperature of 50 °C. The sensor performance was investigated for various gases such as H_2_, CH_4_, H_2_S and CO_2_ at different operating temperatures from 50 °C to 175 °C for various gas concentrations ranging from 7 ppm to 10,000 ppm (1%). The sensor is highly efficient as it detects hydrogen even at low concentration of ~7 ppm and at operating temperature of 50 °C. The sensor’s minimum limit of detection and relative response at 175 °C were found 7 ppm with ~38.7% for H_2_, 110 ppm with ~6.08% for CH_4_, 500 ppm with ~10.06% for H_2_S and 1% with ~11.87% for CO_2_. Here, Pd exhibits dual characteristics as metal contact and excellent catalyst to hydrogen molecules. The activation energy was calculated for all the gases and found lowest ~3.658 kJ/mol for H_2_. Low activation energy accelerates desorption reactions and enhances the sensor’s performance.

## Introduction

All living organisms in an ecosystem pertain to extreme consequences through the confined atmosphere, hence it is mandatory to monitor the level of gases and subsequently their effect on climate. Hydrogen is one of the explosive gas and have huge potential applications such as reactant in hydrogenation for sulphur separation and nitrogen compounds in oil refinery, propulsion fuel of space flight, coolant in electrical generators in power plant, generation of ammonia for fertilizer industry and growth of epitaxial silicon from silicon tetrachloride^1–3^. Due to low ignition energy (0.02 mJ) and broad explosive range (4–75%) of hydrogen, safety precaution is highly recommended^4, 5^. High sensitivity, low power consumption, high selectivity and low cost are essential aspects of hydrogen sensor to detect leakages in industry as well as domestic sector^6–9^. Currently, resistive gas sensors are broadly employed due to their good performance, low cost and compatibility with electronic circuits^10^. However, the resistive gas sensors have some deficiencies such as poor selectivity and high operating temperature, which requires further advancement of these gas sensors^11^. To fabricate a highly selective sensor is still a prime task for researchers. Generally, a wide range of gases can interact with chemisorbed oxygen ions on to the surface of sensing layer and subsequently may create interference to the gas analyte which has to be detected surrounded by other gases. Thus, to prevent from the false signal of unwanted gas species, many reports have been published to rectify the issue of cross sensitivity in order to obtain highly selective sensor response by using various techniques such as (a) doping of transition metal into metal oxide^12–14^, (b) decoration of noble metal nanoparticles over the metal oxide^15, 16^, (c) employment of hybrid metal oxide sensing layers^17, 18^, (d) altering the operating temperature^19, 20^ and (e) use of filtering layers^21–23^. However, altering the operating temperature of sensing layer is more effective method to increase selectivity because of difference in adsorption energy and surface reactivity of various gas species but it also has some adverse effect such as the changing in grain size or surface structure of sensing layer due to higher temperature.

ZnO an n-type semiconductor, belongs to a direct band gap of 3.37 eV^24^. It has tremendous properties like high chemical and thermal stability and large exciton binding energy (60 meV)^25^. ZnO is also used for various gas detection such as H_2_, NO_2_, CH_4_, CO_2_, H_2_S, NH_3_ and volatile organic compounds. The ZnO based gas sensor’s behaviour was highly influenced by surface morphology, dopant and operating temperature. 1-D nanostructures such as nanotubes, nanorods (NRs), nanowires and nanoneedles shows high sensitivity than thin films because of high aspect ratio and higher diffusion of gases^26–28^. Resistive gas sensors with noble metals such as Pd, Pt and Au exhibit superior sensing response for hydrogen gas due to their catalytic activity and solubility of hydrogen atom^29, 30^. Among them, Pd has been most studied in hydrogen sensor owing to its excellent capability of adsorption of hydrogen molecules and their dissociation into hydrogen atoms. Moreover, the diffusion of hydrogen atoms is accelerated throughout the surface and leads to increase the interaction with active sites of the surface^31^. Basu *et al.* has fabricated ZnO thin films based sensor (Pd/ZnO/p-Si and Pd/ZnO/Zn) and found highly hydrogen selective sensor in the range of 2000–20000 ppm H_2_ concentration^32^. Lupan *et al.* fabricated single Cd doped ZnO nanowire using focused ion beam and H_2_ was detected down to 100 ppm at room temperature with good sensitivity while poor response to CH_4_, C_2_H_5_OH, O_2_, LPG and NH_3_^33^. Mondal *et al.* synthesized the ZnO-SnO_2_ composite type hydrogen sensor for different concentration of tested gases at 150 °C and the cross sensitivity of this sensor to CH_4_ and CO was not high at same temperature^34^. Ren *et al.* has prepared ZnO nanowires networks decorated with photo-decomposed Pd nanoparticles by CVD and obtained highly selective sensor response to 1000 ppm H_2_ whereas C_2_H_5_OH, (CH_3_)_2_CO, NO_2_, HCHO and NH_3_ displayed the poor sensitivity^35^. Hong *et al.* reported highly selective PMMA-coated Pd nanoparticles/single-layer graphene hybrid sensor for H_2_, owing to the selective H_2_ filtration effect of the PMMA^23^. Many reports are available on hydrogen sensing by Pd nanoparticles decoration on the ZnO nanostructures, but only few reports are available by Pd contacted ZnO. Hence this work is carried out to explore further performance of Pd contacted ZnO nanorods based hydrogen sensor. Pd contacted vertically aligned ZnO NRs based sensors were fabricated by RF magnetron sputtering technique. Sensor’s response were characterized in presence of various gases such as H_2_, CH_4_, H_2_S and CO_2_ at various concentration and operating temperature ranging from 50 °C to 175 °C. Gas sensing mechanism based on activation energy is discussed to explain high selectivity towards hydrogen gas in comparison to other gases.

## Results

**Figure 1:**
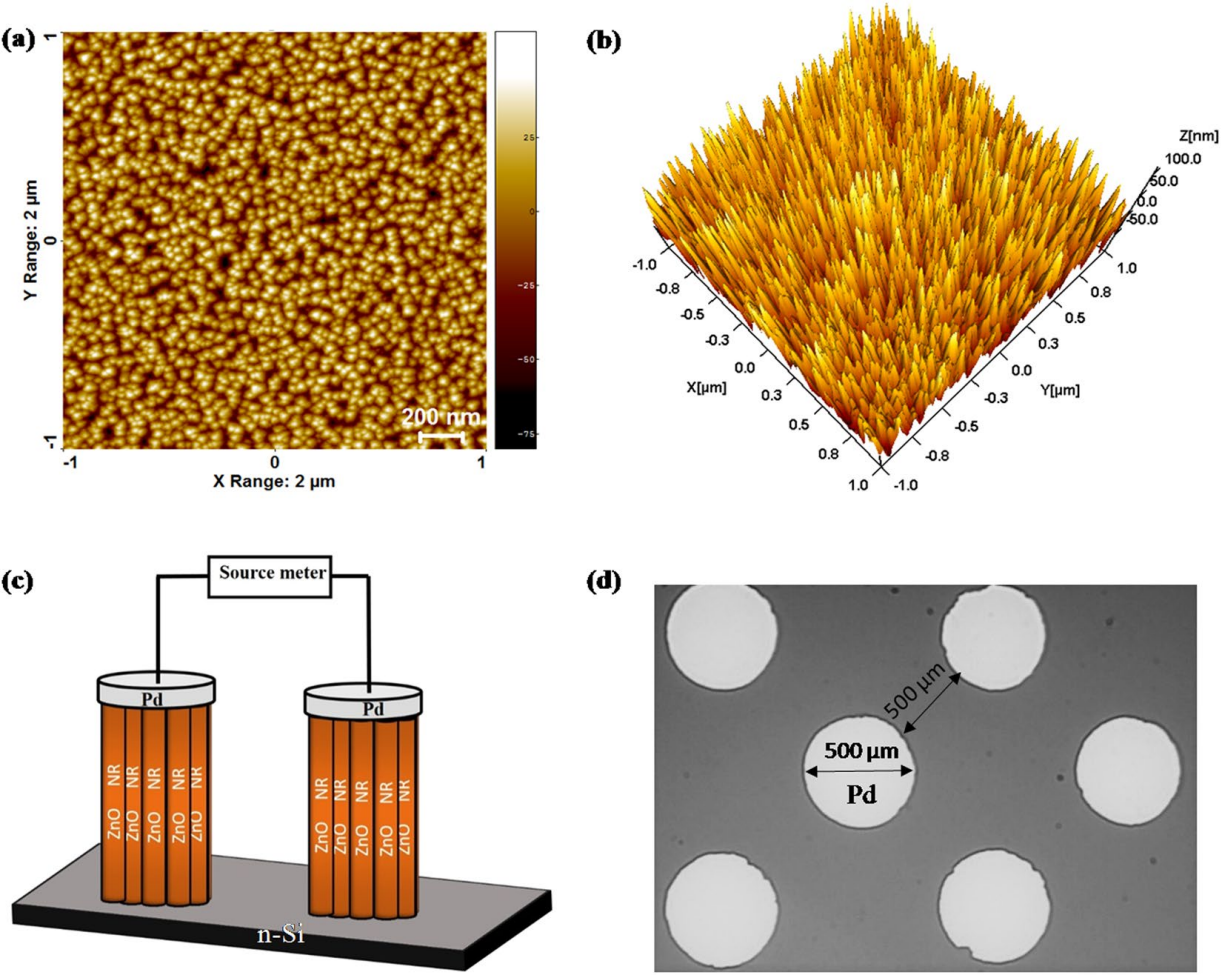
(**a,b**) 2-D and 3-D AFM images of ZnO NRs; (**c,d**) The schematic diagram and optical microscope image of the device.

**Figure 2:**
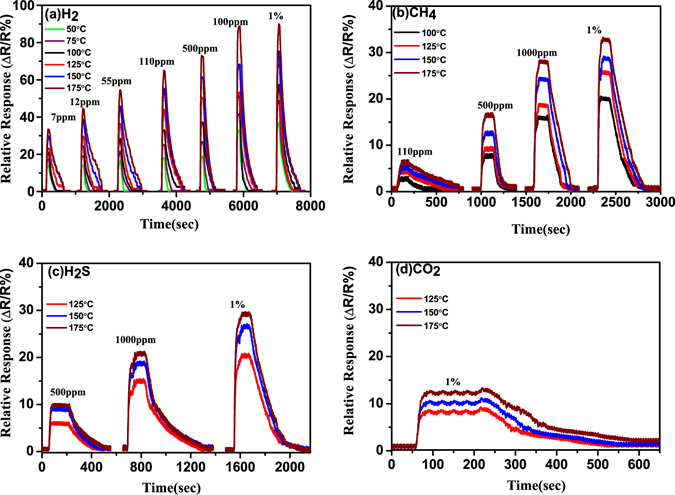
Sensor’s relative response curve with time for 7 ppm to 10,000 ppm of gases: (**a**) H_2_, (**b**) CH_4_, (**c**) H_2_S and (**d**) CO_2_, at operating temperature ranging from 50 °C to 175 °C.

**Figure 3:**
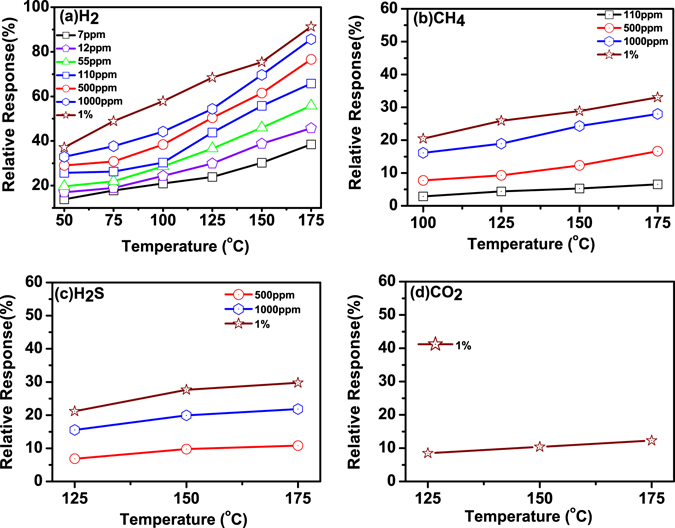
Sensor’s relative response variation with operating temperature and gas concentration.

**Figure 4:**
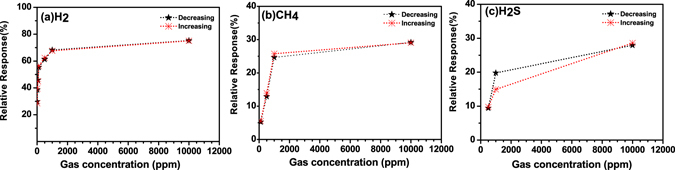
(**a–c**) Hysteresis studies for H_2_, CH_4_ and H_2_S at 150 °C for increasing and decreasing gases concentration.

ZnO NRs were uniformly grown on Si substrate and AFM images are shown in Fig. 1(a,b). Nanorods are highly crystalline and grown along c-axis which is reported in our previous study^36^. The schematic diagram and top view of the device are shown in Fig. 1(c,d). The sensor’s performance depends on chemisorption reaction of reactive gases with adsorbed oxygen ions on ZnO NRs surface. The performance of Pd contacted ZnO NRs based sensor may be influenced by gas type, concentrations and operating temperatures. The sensor’s relative response has been studied for various gases such as H_2_, CH_4_, H_2_S and CO_2_ at different concentrations and operating temperatures. Figure 2 shows sensor’s relative response curve with time for 7 ppm, 12 ppm, 55 ppm, 110 ppm, 500 ppm, 1000 ppm and 10,000 ppm (1%) concentrations of gases (a) H_2_, (b) CH_4_, (c) H_2_S and (d) CO_2_ at operating temperature ranging from 50 °C to 175 °C. The response of the sensor increases with hydrogen concentration and temperature. It was found that the sensor is able to detect very low hydrogen concentration of ~7 ppm and at low temperature (50 °C). The relative response was measured 13.86% at 50 °C and 38.47% at 175 °C. The change in relative response increases with operating temperature due to creating more chemisorbed oxygen ions on the ZnO NRs surface which further enhances desorption of oxygen. The sensor response increases from 13.86% to 37.13% with concentration from 7 to 10,000 ppm and operating temperature 50 °C. A large variation in sensor’s response is observed with low concentration below 1000 ppm since hydrogen molecules react with adsorbed oxygen ions and decrease the depletion region as well as reduce the barrier height at Pd/ZnO junction. Above 1000 ppm of hydrogen chemisorbed reaction is almost saturated on ZnO NRs which is shown by very less increase in sensor’s response from 86.39% to 91.26% at 175 °C of operating temperature. To study the selectivity the relative response of the device was measured for various gases such as CH_4_, H_2_S and CO_2_ with different concentration from 7 ppm to 1%. Figure 2b shows relative response curve with time of CH_4_ gas and minimum detection limit was found 110 ppm at 100 °C. During loading/deloading of the gas, methane gas molecules react with adsorbed oxygen ions on ZnO NRs surface and show decrease/increase of ZnO NRs resistance, which is similar to hydrogen gas behaviour. Although CH_4_ gas is highly stable below 100 °C and with increasing operating temperature above this limit, C-H bonds starts breaking and it gives more H atoms to react with adsorbed oxygen ions and decreases sensor’s resistance. Furthermore, the relative response of H_2_S gas is shown in Fig. 2c and minimum detection limit was observed 500 ppm at 125 °C and maximum relative response is around 30% for 1%. This indicates less reactivity of H_2_S molecules with adsorbed oxygen in comparison to hydrogen and methane gases. Sensor’s relative response was also measured with CO_2_ gas and shown in Fig. 2d. The minimum concentration was detected 1% at 125 °C, because of its highest stable nature in comparison to other reactive gases. Hence, the sensor shows high relative response as well as high selectivity for hydrogen gas in comparison to other gases. Sensor’s relative response variation with operating temperature and concentration is shown in Fig. 3 for (a) H_2_, (b) CH_4_, (c) H_2_S and (d) CO_2_. Figure 4(a–c) depicts hysteresis studies for H_2_, CH_4_ and H_2_S at 150 °C for increasing and decreasing gas concentrations. A stable behaviour of the sensors has been observed for each target gas.

## Discussion

**Figure 5:**
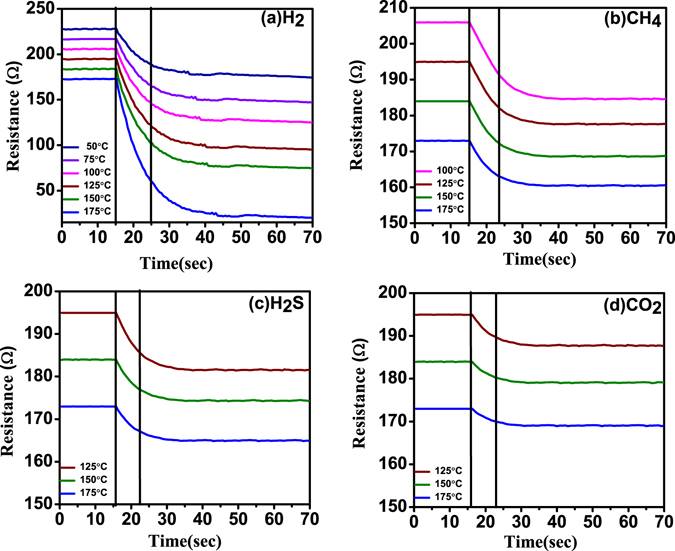
Resistance verses time curve for (**a**) H_2_, (**b**) CH_4_, (**c**) H_2_S and (**d**) CO_2_ gases with 1% concentration at various temperatures.

**Figure 6:**
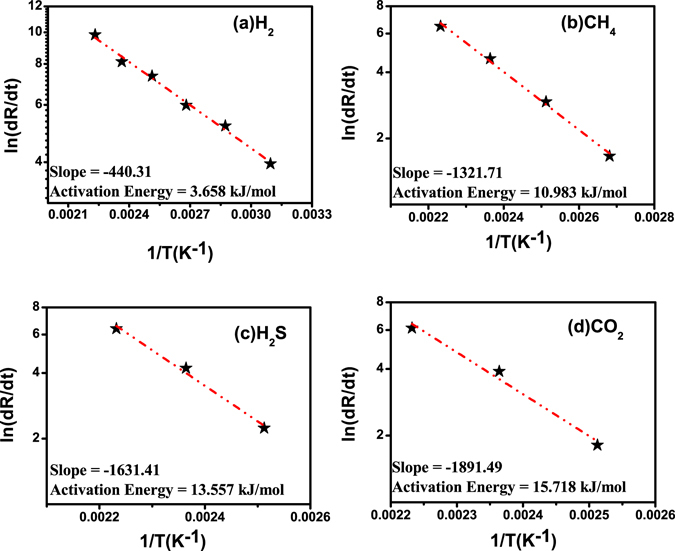
Arrhenius plot of rate of resistance change with temperature after exposure of 1% concentration of (**a**) H_2_, (**b**) CH_4_, (**c**) H_2_S and (**d**) CO_2_ gases.

**Figure 7:**
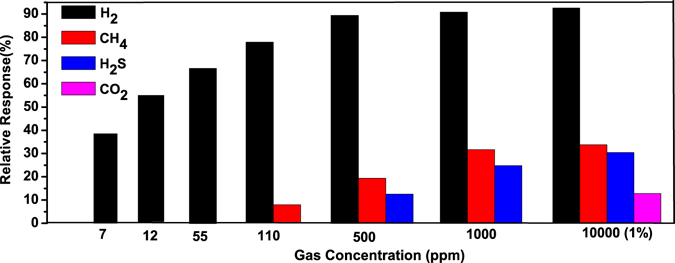
The selectivity histogram of the sensor to H_2_, CH_4_, H_2_S and CO_2_ at 175 °C operating temperature.

**Figure 8:**
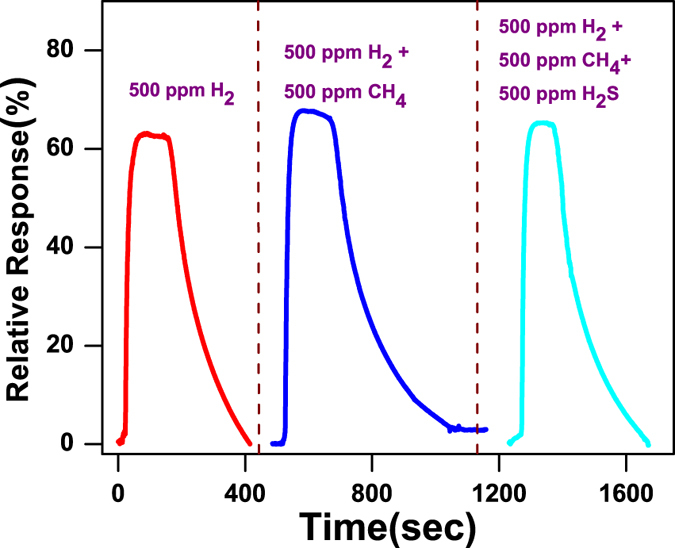
Cross selectivity response curve for various gases mixture (500 ppm H_2_, 500 ppm H_2_ + 500 ppm CH_4_ and 500 ppm H_2_ + 500 ppm CH_4_ + 500 ppm H_2_S) at 150 °C operating temperature.

Gas sensing mechanism is basically surface phenomenon. The fast adsorption-desorption cycle of chemisorbed oxygen on the surface of ZnO nanorods is highly desirable to make excellent gas sensor. Thus surface sensing properties for various gases could be understood by activation energy phenomenon. The rate of resistance changes with respect to temperature for particular target gas may be assumed by Arrhenius equation which can be expressed as^37^:

1 $$R={R}_{o}\exp (\frac{{\rm{\Delta }}E}{KT})$$

where R is resistance of the sensor, K is the Boltzmann constant, T is absolute temperature and ΔE is the activation energy. For activation energy calculation, firstly the rate of change in resistances were evaluated from resistance verses time curves on exposure of (a) H_2_, (b) CH_4_, (c) H_2_S and (d) CO_2_ gases with 1% concentration at different operating temperature and shown in Fig. 5(a–d). It can be seen that the resistance of the sensor is decreased drastically with increase in temperature for hydrogen gas as compared to the other reactive gases. Arrhenius Plot of rate of resistance change with temperature after exposer of 1% concentration of (a) H_2_, (b) CH_4_, (c) H_2_S and (d) CO_2_ gases are shown in Fig. 6(a–d). The activation energy has been evaluated from slope of Arrhenius plot which is basically linear fitting of resistance rate with respect to temperature. The activation energy was calculated as 3.658 kJ/mol, 10.983 kJ/mol, 13.556 kJ/mol and 15.718 kJ/mol for H_2_, CH_4_, H_2_S and CO_2_ gas, respectively. The activation energy is lowest for hydrogen gas as compared to other reactive gases. Our calculated activation energy of Pd contacted ZnO NRs is smaller as compared to Wang *et al.* reported 11.8 kJ/mol for hydrogen sensor based on cluster of Pd/multiple ZnO nanorods^38^. The selectivity histogram of the sensor is shown in Fig. 7 for different concentration at 175 °C operating temperature. The sensor is able to detect minimum 7 ppm hydrogen concentration with ~38.47% relative response at 50 °C. However, for other gases minimum gas concentration sensing limits is quite high in comparison to hydrogen gas. As we know that hydrogen is smallest molecule with lowest activation energy which enhances surface reaction and corresponds to large change in depletion region of ZnO NRs. The minimum detected concentration for CH_4_ gas was observed 110 ppm with 6.5% sensor response which is 10.11 times less than hydrogen gas response (~65.75). H_2_S gas has quite high activation energy (13.556 kJ/mol) in comparison to both Hydrogen and methane gas, shows minimum sensing concentration to 500 ppm with relatively low sensor response ~10.82%. These sensor response is around 7 times less than hydrogen response (~76.56%) for 500 ppm concentration. CO_2_ is highly stable gas with highest activation energy which makes it least reactive gas in comparison to other gases. For CO_2_, minimum gas detection limit was 1% with sensor response ~12.29% which is 7.42 times less than hydrogen response (~91.26%). Cross selectivity has been calculated for Pd/ZnO nanorods based sensor to acquire more clarity for better selectivity. Figure 8 depicts cross selectivity response curve for various gases mixture (500 ppm H_2_, 500 ppm H_2_ + 500 ppm CH_4_ and 500 ppm H_2_ + 500 ppm CH_4_ + 500 ppm H_2_S) at 150 °C operating temperature. It is found that mixing H_2_ gas with other gases with same concentration, there is slight increase in sensor’s response in comparison to pure hydrogen gas. This result implies that Pd/ZnO nanorods based sensor shows high selectivity towards hydrogen gas.

### Gas sensing mechanism

**Figure 9:**
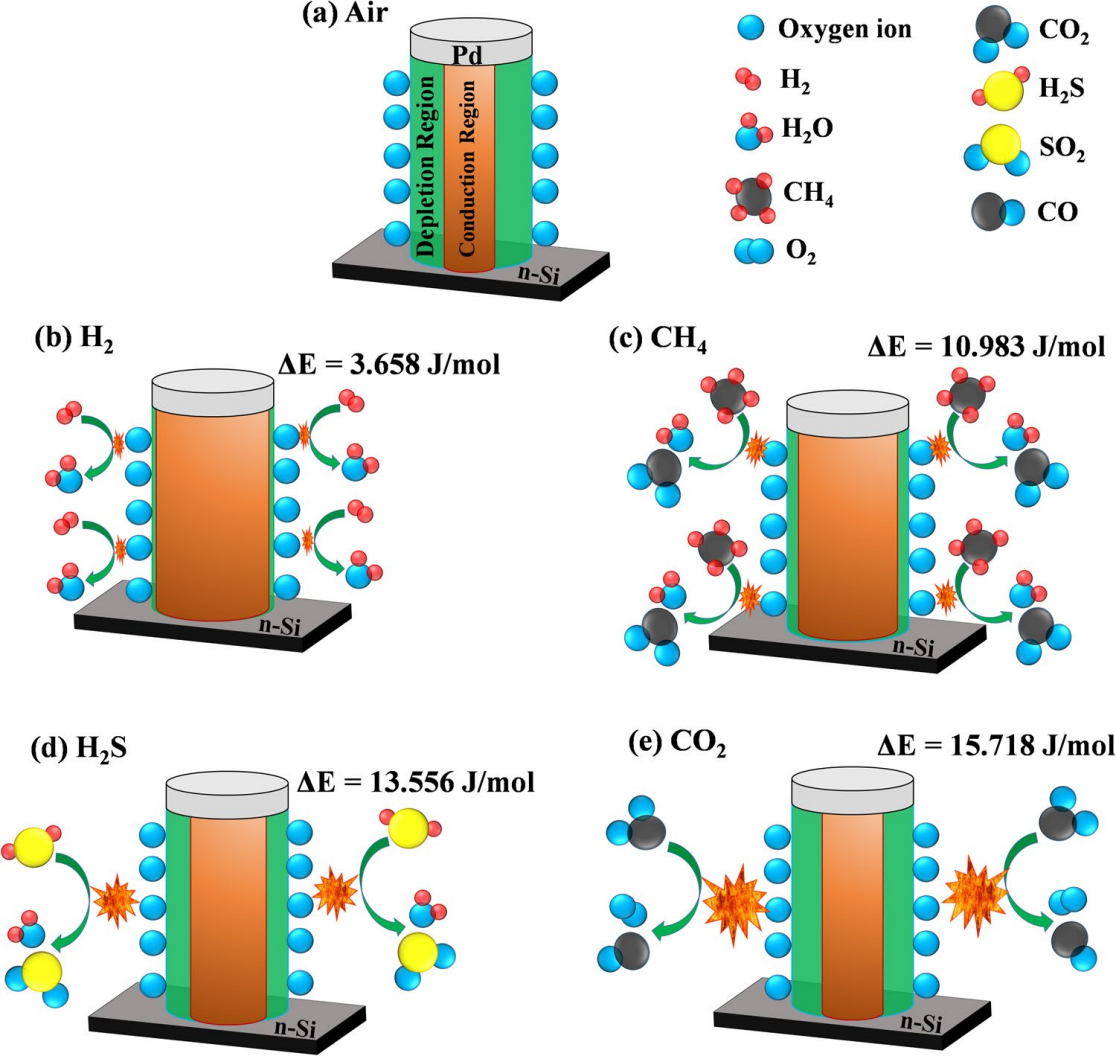
The gas sensing mechanism for sensor; a relative change in depletion region of ZnO NRs in presence of (**a**) Air, (**b**) H_2_, (**c**) CH_4_, (**d**) H_2_S and (**e**) CO_2_ gases.

Based on experimental investigation a gas sensing mechanism is proposed and shown in Fig. 9. From the figure, the relative change in depletion region of ZnO can be seen in presence of (a) Air, (b) H_2_, (c) CH_4_, (d) H_2_S and (e) CO_2_. The sensing mechanism is mainly influenced by activation energy of target gases on the surface of ZnO nanorods and a change in barrier height between Pd and ZnO NRs. When sensor chamber is loaded with target gas, these target gas molecule reacts with adsorbed oxygen ions. By removing chemisorbed oxygen ions from ZnO nanorods surface, trapped electron moves back to conduction region and decreases depletion region width of ZnO nanorods to a large extent^39–41^ as shown in Fig. 9(b–e). For n-type semiconductor materials, CO_2_ gas molecules also react with adsorbed oxygen ions and returns excess electrons to material. These reactions causes decreased sensor’s resistance while loading of CO_2_ gas^42^. Due to increasing activation energy of CH_4_, H_2_S and CO_2_ gases in comparison to H_2_, maximum sensor response is observed for hydrogen at even ppm level with maximum change in depletion region. As activation energy of target gases increases, sensor response decreases with relatively small change in depletion region. These results clearly depict gas sensing mechanism schematic in Fig. 9(b–e). Along with activation energy, operating temperature and target gas concentration also plays an important role in sensor’s response. With increasing temperature, chemisorption of oxygen ions on ZnO nanorods surface increases as conduction band electron get sufficient energy to overcome barrier. Due to these enhanced chemisorbed oxygen ions, there is large change in depletion region as well as in sensors resistance. While loading with target gases, it enhances desorption of oxygen ions due to which relative change in sensor resistance increases. With increasing gas concentration, it provides large reaction with adsorbed oxygen ions which further enhances sensor’s response.

In case of hydrogen, the dissociation of the molecules over the Pd contact is faster and diffuse the hydrogen atom to the interface due to smallest molecular size^23, 43^. Moreover, the conduction of ZnO nanorods is correlated with cross section and given as follow^44^,

2 $${\rm{G}}=\frac{{\rm{1}}}{{\rm{R}}\,}=\frac{{\rm{A}}}{{\rho }{\rm{l}}}=\frac{\mathrm{ne}{\mu }{\rm{A}}}{{\rm{l}}}$$

where G, n, e, l, and µ are electrical conductivity, electron concentration, length of NR and electron mobility, respectively. Fast oxygen desorption will take place when ZnO NRs are exposed to hydrogen and increase the conduction cross section channel area. When Pd is exposed to hydrogen gas, hydrogen molecule gets diffused into Pd which reduces Schottky barrier to a large extent. Because of Schottky barrier reduction along with depletion region reduction, the combine effect maximizes sensors response and makes ppm level detection possible. However as compared with hydrogen molecules, other target gases such as CH_4_ and H_2_S gases are less reactive to Pd contact and slower diffusion due to larger molecular weight and size. Because of these factors, there is a less change in Schottky barrier reduction which causes lesser sensor response in comparisons to hydrogen gas. While relative response of CO_2_ gas is lowest occurred by the (i) more activation energy is required to break double bond than single bond (ii) highest molecular weight as compared to H_2_, CH_4_ and H_2_S and (iii) highest activation energy of CO_2_ gas provides the small relative response.

## Conclusion

We have demonstrated Pd contacted ZnO NRs based sensor which shows high sensor sensitivity and selectivity for hydrogen. The sensor was analysed for selectivity test towards H_2_, CH_4_, H_2_S and CO_2_ and found best suited for H_2_ even at extreme low concentration (~7 ppm) and low operating temperature 50 °C. Because of lowest activation energy ~3.658 kJ/mol of hydrogen in comparison to other reactive gases, it leads to maximum change in conduction cross section area of ZnO NRs which could be possible cause for excellent sensor’s performance. A poor sensitivity was detected for CH_4_ at 110 ppm (~6.5% response) which is 10.11 times less than H_2_ (~65.75% response), for H_2_S gas at 500 ppm (~10.82% response) which is 7 times less than H_2_ (~76.56% response) and for CO_2_ at 1% (~12.29% response) and marked 7.42 times less than H_2_ (~91.26%). The gas sensing mechanism for Pd contacted ZnO NRs based sensor was also proposed which mainly attributed to activation energy of reactive gases. The proposed sensor is not only highly hydrogen selective but also can operate at low temperature which reduces the power consumption and prove to be energy efficient in its class.

## Experimental Techniques

Vertically aligned ZnO NRs were fabricated on n-Si substrate using RF sputtering. Substrate temperature was kept constant at 600 °C during the deposition and other parameters can be found elsewhere^36^. Uniform distribution of ZnO NRs was confirmed by using AFM (Park XE-70) and FESEM techniques. For sensor fabrication, circular palladium (Pd) electrodes of 500 µm diameter were deposited on ZnO NRs with the help of DC Sputtering technique where shadow mask was used. Thickness of Pd electrodes was kept around 150 nm and 500 µm inter-electrode spacing. Gas sensing characterization was carried out in a stainless steel sensing chamber. The chamber was evacuated down to 3 × 10^−3^ mbar using a rotary pump. The testing gases were mixed with argon and introduced in the sensing chamber by injecting a pre-calibrated volume of target gas through mixing chamber and a gas tight syringe (Hamilton). Operating temperature was tuned from 50 °C to 175 °C. The sensor’s response was measured by Semiconductor Parameter analyser (Keithey 4200-SCS) by applying a constant voltage of 1 V.

## References

[1] Schlapbach L, Zuttel A (2001). Hydrogen-storage materials for mobile applications. Nature.

[2] Züttel A (2003). Materials for hydrogen storage. Mater. Today.

[3] Ramchandran R, Menon RK (1998). An overview of industrial uses of hydrogen. Int. J. Hydrogen Energy.

[4] Sakintuna B, Lamari-Darkrim F, Hirscher M (2007). Metal hydride materials for solid hydrogen storage: a review. Int. J. Hydrogen Energy.

[5] Jung D, Han M, Lee GS (2014). Room-temperature gas sensor using carbon nanotube with cobalt oxides. Sens. Actuators B.

[6] Zhang P, Vincent A, Kumar A, Seal S, Cho HJ (2010). A Low-Energy Room-Temperature Hydrogen Nanosensor: Utilizing the Schottky Barriers at the Electrode/Sensing-Material Interfaces. IEEE Electron Device Lett.

[7] Ozgur U (2005). A comprehensive review of ZnO materials and devices. J. Appl. Phys..

[8] Ma N, Suematsu K, Yuasa M, Kida T, Shimanoe K (2015). Effect of water vapor on Pd-loaded SnO_2_ nanoparticles gas sensor. ACS Appl. Mater. Interfaces.

[9] Liu B, Cai D, Liu Y, Wang D, Wang L, Wang Y (2014). Improved room-temperature hydrogen sensing performance of directly formed Pd/WO_3_ nanocomposite. Sens. Actuators B.

[10] Gu H, Wang Z, Hu Y (2012). Hydrogen gas sensors based on semiconductor oxide nanostructures. Sensors.

[11] Xu M, Li Q, Ma Y, Fan H (2014). Ni-doped ZnO nanorods gas sensor: Enhanced gas-sensing properties, AC and DC electrical behaviors. Sens. Actuators B.

[12] Li YJ, Li KM, Wang CY, Kuo CI, Chen LJ (2012). Low-temperature electrodeposited Co- doped ZnO nanorods with enhanced ethanol and CO sensing properties. Sens. Actuators B.

[13] Cheng JP, Wang BB, Zhao MG, Liu F, Zhang XB (2014). Nickel-doped tin oxide hollow nanofibers prepared by electrospinning for acetone sensing. Sens. Actuators B.

[14] Lin Z, Li N, Chen Z, Fu P (2017). The effect of Ni doping concentration on the gas sensing properties of Ni doped SnO_2_. Sens. Actuators B.

[15] Agar P, Mehta BR, Varandani D, Prasad AK, Kamruddin M, Tyagi AK (2010). Sensing response of palladium nanoparticles and thin films to deuterium and hydrogen: Effect of gas atom diffusivity. Sens. Actuators B.

[16] Sanger A, Kumar A, Kumar A, Chandra R (2016). Highly sensitive and selective hydrogen gas sensor using sputtered grown Pd decorated MnO_2_ nanowalls. Sens. Actuators B.

[17] Katoch A, Kim JH, Kwon YJ, Kim HW, Kim SS (2015). Bifunctional Sensing Mechanism of SnO_2_–ZnO Composite Nanofibers for Drastically Enhancing the Sensing Behavior in H_2_ Gas. ACS Appl. Mater. Interfaces.

[18] Li C (2015). Electrospun nanofibers of p-type NiO/n-type ZnO heterojunction with different NiO content and its influence on trimethylamine sensing properties. Sens. Actuators B.

[19] Balouria V (2011). Temperature dependent H_2_S and Cl_2_ sensing selectivity of Cr_2_O_3_ thin films. Sens. Actuators B.

[20] Hastir A, Kohli N, Singh RC (2016). Temperature dependent selective and sensitive terbium doped ZnO nanostructures. Sens. Actuators B.

[21] Fleischer M, Seth M, Kohl CD, Meixner H (1996). A selective H_2_ sensor implemented using Ga_2_O_3_ thin-films which are covered with a gas-filtering SiO_2_ layer. Sens. Actuators B.

[22] Kwon CH (2000). Multi-layered thick-film gas sensor array for selective sensing by catalytic filtering technology. Sens. Actuators B.

[23] Hong J, Lee S, Seo J, Pyo S, Kim J, Lee T (2015). A Highly Sensitive Hydrogen Sensor with Gas Selectivity Using a PMMA Membrane-Coated Pd Nanoparticle/Single-Layer Graphene Hybrid. ACS Appl. Mater. Interfaces.

[24] Jia X, Fan H, Afzaal M, Wu X, Brien PO (2011). Solid state synthesis of tin doped ZnO at room temperature: characterization and its enhanced gas sensing and photocatalytic properties. J. Hazard. Mater..

[25] Dar GN (2012). Ce-doped ZnO nanorods for the detection of hazardous chemical. Sens. Actuators B.

[26] Mani GK, Rayappan JBB (2014). Selective detection of ammonia using spray pyrolysis deposited pure and nickel doped ZnO thin film. App. Sur. Sci.

[27] Wang X, Zhao M, Liu F, Jia J, Li X, Cao L (2013). C_2_H_2_ gas sensor based on Ni -doped ZnO electrospun nanofibers. Ceramics International.

[28] Bouaoud A (2013). Transparent conducting properties of Ni doped zinc oxide thin films prepared by a facile spray pyrolysis technique using perfume atomizer. Mat. Chem. Phys..

[29] Hübert T, Boon-Brett L, Black G, Banach U (2011). Hydrogen sensors - A review. Sens. Actuators B.

[30] Pundt A (2004). Hydrogen in Nano-sized Metals. Adv. Eng. Mater..

[31] RaviPrakash J (2007). Hydrogen sensors: Role of palladium thin film morphology. Sens. Actuators B.

[32] Basu S, Dutta A (1997). Room-temperature hydrogen sensors based on ZnO. Mater. Chem. Phy.

[33] Lupan O (2012). Highly sensitive and selective hydrogen single-nanowire nanosensor. Sens. Actuators B.

[34] Mondal B, Basumatari B, Das J, Roychaudhury C, Saha H, Mukherjee N (2014). ZnO–SnO_2_ based composite type gas sensor for selective hydrogen sensing. Sens. Actuators B.

[35] Ren S, Fan G, Qu S, Wang Q (2011). Enhanced H_2_ sensitivity at room temperature of ZnO nanowires functionalized by Pd nanoparticles. J. Appl. Phys..

[36] Ranwa S, Kumar M, Singh J, Fanetti M, Kumar M (2015). Schottky-contacted vertically self-aligned ZnO nanorods for hydrogen gas nanosensor applications. J. Appl. Phys..

[37] Song P (2007). Characteristics and sensing properties of La_0.8_Pb_0.2_Fe_1−x_Ni_x_O_3_ system for CO gas sensors. Mater. Sci. Eng. B.

[38] Wang HT (2005). Hydrogen-selective sensing at room temperature with ZnO nanorods. Appl. Phys. Lett..

[39] Lupan O, Chai G, Chow L (2008). Novel hydrogen gas sensor based on single ZnO nanorod. Micro. Engineering.

[40] Tshabalala ZP, Shingange K, Dhonge BP, Ntwaeaborwa OM, Mhlongo GH, Motaung DE (2017). Fabrication of ultra-high sensitive and selective CH_4_ room temperature gas sensing of TiO_2_ nanorods: Detailed study on the annealing temperature. Sens. Actuators B.

[41] Hamedani NF, Mahjoub AR, Khodadadi AA, Mortazavi Y (2011). Microwave assisted fast synthesis of various ZnO morphologies for selective detection of CO, CH_4_ and ethanol. Sens. Actuators B.

[42] Jeong YJ, Balamurugan C, Lee DW (2016). Enhanced CO_2_ gas-sensing performance of ZnO nanopowder by La loaded during simple hydrothermal method. Sens. Actuators B.

[43] Zheng ZQ, Zhu LF, Wang B (2015). In_2_O_3_ Nanotower Hydrogen Gas Sensors Based on Both Schottky Junction and Thermoelectronic Emission. Nano. Res. Lett.

[44] Ranwa S, Kulriya PK, Sahu VK, Kukreja LM, Kumar M (2014). Defect-free ZnO nanorods for low temperature hydrogen sensor applications. Appl. Phys. Lett..

